# Impact of Pain and Discomfort on the Lives of Cognitively Impaired Older Adults and Their Informal Caregivers

**DOI:** 10.1093/geroni/igaa057.2848

**Published:** 2020-12-16

**Authors:** Hyunjin Noh

**Affiliations:** The University of Alabama, Tuscaloosa, Alabama, United States

## Abstract

This qualitative study explored the impact of pain and discomfort on the lives of cognitively impaired older adults and their caregivers from the caregiver perspective. Forty-three individuals of age 19+, who identified themselves as primary caregiver to a chronically or seriously ill older adult (age 50+) with cognitive impairment, such as Alzheimer’s Disease and Related Dementia, were recruited at various community settings. Individual, face-to-face interviews were conducted to ask participants how they thought their care-recipient’s pain and discomfort affected the care-recipient’s and the caregiver’s life respectively. Inductive, thematic analysis of interview transcripts revealed several key themes: compromised mobility, limited social interaction or activities, and depressive symptoms in both care-recipients and caregivers; aggravated cognitive decline in care-recipients; and poorer physical health in caregivers. Participants wanted more information on the disease trajectory and available services, particularly home-based therapies and social activities for care-recipients, which provides future program/practice implications.

